# Management of functional outcomes after radical prostatectomy in the Nordic countries: A survey of uro-oncological centers

**DOI:** 10.1038/s41443-023-00772-8

**Published:** 2023-10-10

**Authors:** Alexander Bjørneboe Nolsøe, Henriette Veiby Holm, Teemu J. Murtola, Peter Busch Østergren, Mikkel Fode

**Affiliations:** 1 Department of Clinical Medicine, Faculty of Health and Medical Sciences, University of Copenhagen, Copenhagen, Denmark; 2 Department of Urology, Copenhagen University Hospital, Herlev and Gentofte Hospital, Herlev, Denmark; 3 Department of Urology, Oslo University Hospital Rikshospitalet, Oslo, Norway; 4 Faculty of Medicine and Health Technology, University of Tampere, Tampere, Finland; 5 Department of Urology, TAYS Cancer Center, Tampere, Finland

**Keywords:** Quality of life, Preventive medicine

## Abstract

We aimed to describe the clinical practice regarding erectile dysfunction and urinary incontinence after radical prostatectomy in the Nordic countries. A 37-item survey about pre- and post-prostatectomy evaluation and rehabilitation of sexual and urinary function was sent to 42 uro-oncology centers. Reporting was done according to the Checklist for Reporting Results of Internet E-Surveys (CHERRIES). Twenty-seven centers in Denmark (*n* = 6), Norway (*n* = 8), Finland (*n* = 7), and Sweden (n = 6) responded (64.3%). Post-prostatectomy sexual function was evaluated by 25 centers. The majority used validated questionnaires with significant variations across centers. Post-prostatectomy urinary function was evaluated by 24 centers. Again, the majority used validated questionnaires, while 9 centers used objective measures including uroflowmetry, residual urine volume, and pad usage. Twenty-one centers offered sexual rehabilitation and 12 of these described their protocols. All centers administered phosphodiesterase-5 inhibitors and seven centers offered further treatment options. Two centers offered a consultation with a sexologist. Twenty-three centers provided pelvic floor muscle training and one center used medical support with duloxetine. Our study indicates a need for standardized evaluation and management of erectile dysfunction and urinary incontinence following radical prostatectomy. Especially, there is a need for an increased focus on comprehensive sexual rehabilitation.

## Introduction

Radical prostatectomy (RP) is a commonly used curative treatment for localized prostate cancer [1]. Unfortunately, it is associated with two common adverse effects: erectile dysfunction (ED) and urinary incontinence (UI). The incidence of ED after RP ranges from about 25% to 75%, while UI has been reported in 4% to 31% of patients after 1 year of follow-up, depending on the definitions used [2, 3].

In non-nerve sparing surgery, the resection of the cavernous nerves leads to ED [4]. On the other hand, in nerve-sparing surgery, it is believed that a temporary period of neuropraxia occurs after the operation, which, due to the lack of erections, results in insufficient oxygenation and fibrosis in the cavernous tissue, leading to permanent ED [5]. To counteract this, combinations of erectogenic drugs and devices are often used in the postoperative period as penile rehabilitation. Despite the widespread use and belief in its effectiveness [6, 7], results from randomized human trials have been predominantly negative, with a 2018 Cochrane review finding no benefit over placebo [8]. The current EAU guidelines do not make any specific recommendations for penile rehabilitation regimens [9].

The etiology of UI after RP is multifactorial and includes surgical alterations of supporting structures, damage to the urethral sphincter, and damage to the pelvic nerves [10]. Patient factors such as age, BMI, and preexisting lower urinary tract symptoms are also associated with a higher risk of UI [11]. Studies suggest that pre- or post-operative pelvic floor muscle training (PFMT) may reduce incontinence in the short term, but there are conflicting data regarding its long-term impact [12, 13]. The AUA/SUFU guidelines recommend offering PFMT to UI patients as the potential benefits outweigh the risks [14]. The EAU guidelines mention that PFMT appears to speed up continence recovery following RP [15]. Currently, there are no other established continence rehabilitation protocols [16, 17].

Given the lack of consensus and clear guidelines regarding the evaluation and rehabilitation of post-prostatectomy ED and UI, it is important to assess the current clinical practices. Such investigations can stimulate informed discussions about optimal protocols and guide future research in this area. The purpose of this study is to provide a comprehensive description of practices in Nordic uro-oncology centers.

## Materials and methods

We developed a 37-item online survey and sent invitations containing a link to the survey by email to the chairpersons of all 42 uro-oncology centers performing at least 20 radical prostatectomies per year in the Nordic countries. We created and managed the survey using the Research Electronic Data Capture program (REDCap), which is a secure system that automatically captures the responses directly. Survey questions were developed by the research group and adjusted based on internal discussion. It was comprised of simple questions regarding the surgical volume and techniques, as well as on institutional practices regarding evaluation of pre-and post-operative sexual and urinary function, and existence of rehabilitation protocols. If respondents indicated that they evaluated functional outcomes and/or provided rehabilitation, they were asked to detail the timing and methods through open ended write-in questions. The invitations, along with a cover letter explaining the purpose of the study, were distributed via email to facilities in Denmark (*n* = 7), Norway (*n* = 12), Finland (*n* = 8), and Sweden (*n* = 15). The purpose of the study was described, and it was specified which data were stored and who the investigators were. It was allowed for the recipients to delegate the task of filling out the survey to other relevant physicians at their center. Each center was considered a unique responder, and duplicates were avoided as the name of the center was given as a mandatory part of the survey. No cookies or IP address check were used. No incentives were offered for filling out the survey. The survey consisted of initial questions regarding surgical characteristics, such as case volume, surgical approach, and nerve-sparing techniques. Subsequent items explored methods of sexual and urinary evaluation before and after surgery, as well as sexual and urinary rehabilitation practices. The survey was available on one screen and employed adaptive questioning to reduce its complexity. For example, responders were only asked to specify their rehabilitation program if they had indicated that they had one. There was an automatic completeness check before submission, which is a feature of REDCap. However, as descriptions of evaluation methods and rehabilitation protocols included write in responses, the thoroughness of the responses could not be automatically checked and were reviewed subsequently. Responders were able to review and change their answers by scrolling back through the survey. All submitted questionnaires were analyzed.

Initially, the survey was distributed in March 2020, and subsequent reminders were sent to each center. However, due to the COVID-19 pandemic, the response time was extended until April 2021 after which, no further reminders were sent. After the completion of the survey, the data was extracted and analyzed using SPSS Statistics 25. Descriptive statistics were performed, and Fisher’s exact test was used when applicable. Since the study only involved questionnaires and did not involve patients, it was exempt from ethical approvals. Reporting was done according to the Checklist for Reporting Results of Internet E-Surveys (CHERRIES).

## Results

A total of 27 out of 42 centers responded to the survey, yielding a response rate of 64.3%. It was not possible to see if some centers viewed the survey without responding. The response rates for each country were as follows: Denmark, 6 out of 7 centers (85.7%); Finland, 7 out of 8 centers (87.5%); Sweden, 6 out of 15 centers (40%); and Norway, 8 out of 12 centers (66.7%). The annual case volume of radical prostatectomy (RP) surgeries varied across centers, ranging from 20 to 500 surgeries per year, with a median of 150.

### Sexual function

**Fig. 1:**
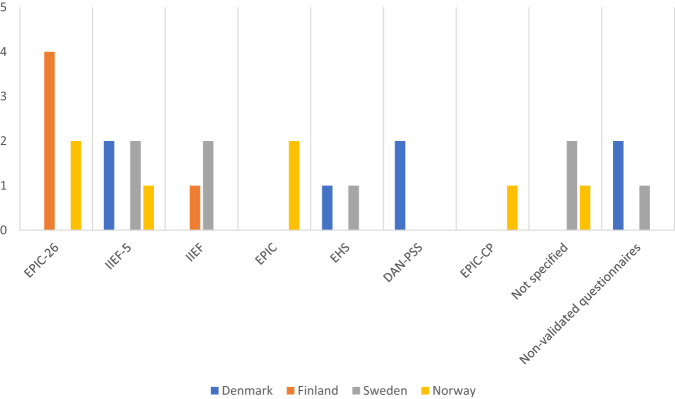
Number of centers using each questionnaire in the pre-operative evaluation of sexual function. EPIC-26 The Expanded Prostate Cancer Index Composite short form; IIEF-5 the IIEF short form; IIEF The International Index of Erectile Function; EPIC The full EPIC questionnaire; EHS The Erection Hardness Scale; DAN-PSS The Danish Prostate Symptom Score; EPIC-CP The EPIC Clinical Practice; Not specified = Centers that replied they used validated questionnaires but did not specify which ones.

**Fig. 2:**
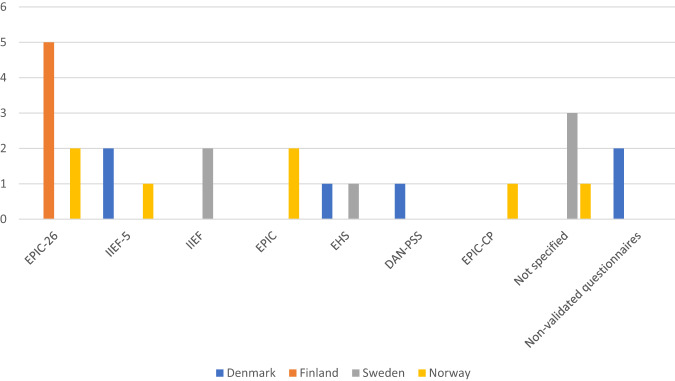
Number of centers using each questionnaire in the post-operative evaluation of sexual function. EPIC-26 The Expanded Prostate Cancer Index Composite short form; IIEF-5 the IIEF short form; IIEF The International Index of Erectile Function; EPIC The full EPIC questionnaire; EHS The Erection Hardness Scale; DAN-PSS The Danish Prostate Symptom Score; EPIC-CP The EPIC Clinical Practice; Not specified Centers that replied they used validated questionnaires but did not specify which ones.

In the preoperative setting, sexual function was evaluated by 25 out of 27 centers (92.6%). Among these centers, 22 out of 25 (88.0%) used questionnaires, and of those, 21 centers (95.5%) employed validated questionnaires including the Expanded Prostate Cancer Index Composite (EPIC) [18], EPIC-26 [19], EPIC-CP [20], the international index of erectile function (IIEF) [21], IIEF-5 [22], the Erection Hardness Scale (EHS) [23], and the Danish Prostate Symptom Score (DAN-PSS) [24]. Five centers used more than one questionnaire. The remaining 3 centers relied solely on a subjective assessment consisting of an unstructured interview with the patient. Details regarding the use of questionnaires are summarized in Table 1 and Fig. 1. In the post-operative setting, one center ceased evaluating sexual function, while another center that did not conduct pre-operative evaluations began assessing it post-operatively. As a result, 25 out of 27 centers (92.6%) performed post-operative sexual evaluations. Among these centers, 21 out of 25 (84.0%) utilized questionnaires, with 20 out of 21 (95.2%) using validated questionnaires. Three centers uded more than one questionnaire (see Table 1 and Fig. 2). The remaining four centers relied solely on subjective assessments. Five centers implemented changes in their assessment methods from pre-operative to post-operative evaluations, such as transitioning from questionnaires to subjective assessments.

**Table 1:** Distribution of sexual questionnaires, pre- and post-operatively.

Preoperative sexual questionnaires	
Combinations of questionnaires used per center, *n* (%)	(*n* = 22)
Validated Questionnaires only	18 (81.8)
Combined Validated- and non-validated Questionnaires	3 (13.6)
Non-validated Questionnaires only	1 (4.5)
The number of questionnaires used per center, *n*	
1 questionnaire	16
2 questionnaires	4
3 questionnaires	1
**Postoperative sexual questionnaires**	
Combinations of questionnaires used per center, *n* (%)	(*n* = 21)
Validated Questionnaires only	18 (85.7)
Combined Validated- and non-validated Questionnaires	2 (9.5)
Non-validated Questionnaires only	1 (4.8)
The number of questionnaires used per center, *n*	
1 questionnaire	18
2 questionnaires	3

Twenty-one centers (21/27, 77.8%) reported to offer sexual rehabilitation. Among these, 18 made it accessible to all patients, whereas the remaining three limited it to patients who had undergone nerve-sparing surgery. Specific rehabilitation protocols were detailed by twelve centers. All centers administered phosphodiesterase-5 inhibitors (PDE5Is) as the initial course of therapy to men who had undergone nerve-sparing RP. One center coupled this primary treatment with the use of a vacuum erection device (VED) from the onset, while another center integrated both VED and penile injection therapy along with the initial treatment. Five centers described additional lines of rehabilitation. Among these, one center solely provided VED, another exclusively used injection therapy, one center integrated both VED and injection therapy, and the last two centers offered rehabilitation involving VED, urethral suppositories, and injection therapy. Distinct protocols for patients following non-nerve-sparing surgery were detailed by two centers, in both cases consisting of penile injection therapy. Only two centers reported offering a consultation with a sexologist as a supplement to the medical treatment. There was a significant association between a higher surgical volume and having a sexual rehabilitation protocol (*p* = *0.038*).

### Urinary function

**Fig. 3:**
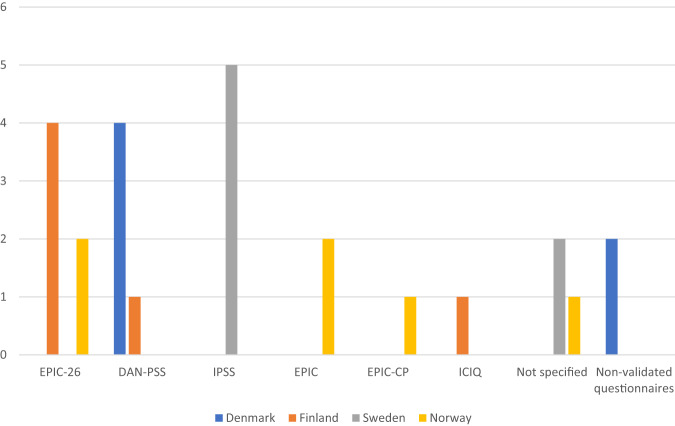
Number of centers using each questionnaire in the pre-operative evaluation of urinary function. EPIC-26 The Expanded Prostate Cancer Index Composite short form; DAN-PSS The Danish Prostate Symptom Score; IPSS The International Prostate Symptom Score; EPIC The full EPIC questionnaire; EPIC-CP The EPIC Clinical Practice; ICIQ The International Consultation on Incontinence Questionnaire; Not specified Centers that replied they used validated questionnaires but did not specify which ones.

The same 25 centers that assessed sexual function before surgery also evaluated urinary function in the preoperative setting. In total, 22 out of 25 centers (88.0%) utilized questionnaires, with 21 out of 22 (95.5%) using validated questionnaires including EPIC, EPIC-26, EPIC-CP, DAN-PSS, The International Prostate Symptom Score (IPSS) [25], and the International Consultation on Incontinence Questionnaire (ICIQ) [26]. Three centers used more than one questionnaire (see Table 2 and Fig. 3). Among these centers, seven (28.0%) combined questionnaires with one or more objective measures, including uroflowmetry, residual urine volume, and daily pad usage. Furthermore, one center relied on a combination of objective assessment and an unstructured patient interview, another center solely employed objective assessments, and yet another center used patient interview only.

**Table 2:** Distribution of questionnaires on urinary function, pre- and post-operatively.

Preoperative urinary evaluation	
Combinations of questionnaires used per center, *n* (%)	(*n* = 22)
Validated Questionnaires only	20 (90.9)
Combined Validated- and non-validated Questionnaires	1 (13.6)
Non-validated Questionnaires only	1 (4.5)
The number of questionnaires used per center, *n*	
1 questionnaire	19
2 questionnaires	3
**Postoperative urinary evaluation**	
Combinations of questionnaires used per center, *n* (%)	(*n* = 19)
Validated Questionnaires only	16 (84.2)
Combined Validated- and non-validated Questionnaires	2 (10.5)
Non-validated Questionnaires only	1 (5.3)
The number of questionnaires used per center, *n*	
1 questionnaire	16
2 questionnaires	3

**Fig. 4:**
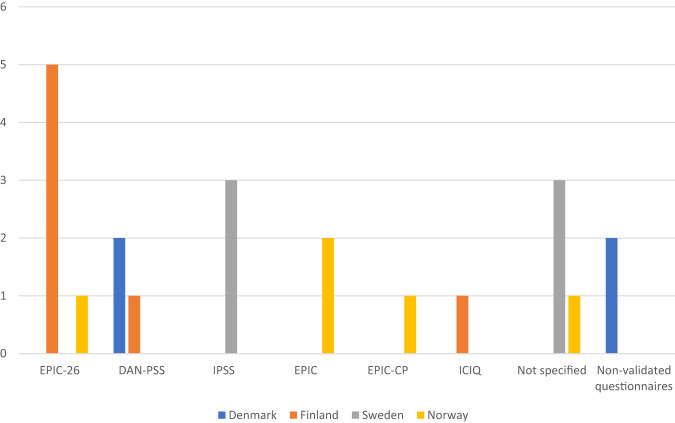
Number of centers using each questionnaire in the post-operative evaluation of urinary function. EPIC-26 The Expanded Prostate Cancer Index Composite short form; DAN-PSS The Danish Prostate Symptom Score; IPSS The International Prostate Symptom Score; EPIC The full EPIC questionnaire; EPIC-CP The EPIC Clinical Practice; ICIQ The International Consultation on Incontinence Questionnaire; Not specified Centers that replied they used validated questionnaires but did not specify which ones.

In the postoperative setting, two centers discontinued evaluating urinary function, while one center that did not assess preoperative urinary function started to do so postoperatively. As a result, 24 out of 27 centers (88.9%) performed urinary evaluations. Among these centers, 19 out of 24 (79.2%) utilized questionnaires, with 17 out of 24 (70.8%) employing the same questionnaires as in the preoperative evaluation. Sixteen out of the 19 centers (84.2%) used validated questionnaires, two combined validated and non-validated questionnaires (10.5%) and one center (5.3%) used a non-validated questionnaire only. Three centers used more than one questionnaire (See Table 2 and Fig. 4). Eight centers (33.3%) combined questionnaires with objective measures. Additionally, one center solely used objective assessments, while four centers relied on subjective assessments.

Twenty-five out of 27 centers (92.6%) offered rehabilitation for UI, with 23 of them providing PFMT through a physiotherapist. Among these centers, 4 out of 23 (17.4%) offered both pre- and post-operative PFMT, while 16 out of 23 (69.6%) provided it to all patients postoperatively. The remaining 3 out of 23 (13.0%) offered PFMT postoperatively only for patients experiencing UI problems. One center employed early medical support with duloxetine, while the last center did not provide a description of its protocol.

There was no statistically significant association between surgical volume and the presence of a continence rehabilitation protocol (p = 0.331).

### Timing of follow-up

Two centers exclusively conducted follow-up evaluations for sexual function at 3, 6, and 12 months, while another center followed up on urinary evaluation at 3 months only. The remaining 23 centers performed follow-up assessments for both sexual function and urinary evaluation during the first prostate-specific antigen (PSA) follow-up visit, which was conducted at 6 weeks (two centers), 2 months (two centers), 3 months (18 centers), to 5 months (one center). The subsequent follow-up visits were scheduled at 6 months (one center), 12 months (16 centers), 18 months (one center), 36 months (one center), 60 months (three centers), and 15 years (one center).

## Discussion

Our study presents the first comprehensive data on the evaluation of sexual and urinary function related to RP and the utilization of rehabilitation protocols in the Nordic countries. We observed significant variations among centers, although most conducted assessments of sexual and urinary function both before and after surgery.

Evaluations were primarily based on validated questionnaires, also known as patient-reported outcome measures (PROMs). These questionnaires assess various functions and their impact on patients’ health-related quality of life. Most PROMs also enable the detection of changes over time [27]. Moreover, the use of validated PROMs allows for comparisons across different study populations. Multiple PROMs were employed to evaluate both urinary and sexual functions. Centers from the same country tended to prefer specific PROMs, but the limited number of centers and the wide range of PROMs used made it difficult to establish statistical significance. No single PROM was utilized by every center within any given country. Interestingly, several centers altered their evaluation methods between the pre-operative and post-operative settings. Such changes introduce a significant risk of misinterpreting results. Even more surprising is the fact that some centers did not assess functional outcomes at all. Establishing recommendations for the optimal evaluation tools and their timing would enhance the consistency of data. This would facilitate more reliable result interpretations and provide a stronger foundation for meta-analyses [28].

Overall, 74% of the centers included in our study offered some form of sexual rehabilitation, and its utilization was associated with a larger surgical volume. Although this percentage is slightly lower compared to findings from other studies, such as 87% of International Society for Sexual Medicine members and 86% of AUA members [6, 7], it is worth noting that our study achieved a much higher response rate at 64.3% compared to the mentioned studies, which had response rates of only 10% and 8%, respectively. This indicates that our study is less susceptible to non-response bias and may provide a more accurate assessment. All sexual rehabilitation protocols in our study employed PDE5Is as the first-line treatment. This choice is not surprising, as PDE5Is are the most straightforward treatment option for ED. However, previous high-quality studies have suggested limited clinical effect in a large proportion of post-RP patients and a lack of effectiveness in terms of rehabilitation [29, 30]. Therefore, it is concerning that only a few centers offered more lines of rehabilitation with VED, urethral suppositories, and injections. These options are often more effective terms of direct treatment effects, although there is a general shortage of high-quality studies assessing their efficacy in rehabilitation [31]. It is of further concern that only a few centers with rehabilitation protocols referred patients to a sexologist. This suggests that despite the limited efficacy of penile rehabilitation, there is still a focus on it rather than taking a holistic approach to addressing patient sexuality. Other physiological methods of sexual rehabilitation, such as penile vibratory stimulation, stretching devices, and low-intensity extracorporeal shock wave therapy, were not utilized in our study, which is reasonable considering the limited available literature [32].

Most centers in our study offered continence rehabilitation, primarily based on PFMT. Although the exact pathophysiology of UI after prostatectomy is not fully understood, there is generally a gradual recovery of urinary continence within the first 12 months after surgery, and most men will regain continence [2]. The theory behind PFMT is that it can strengthen the sphincter mechanism, either by directly targeting the external urethral sphincter or indirectly through its effect on the pelvic floor muscles [33]. However, there is a limited number of high-quality randomized controlled trials (RCTs) in the literature, resulting in heterogeneity in methodology, outcome measures, and differences in PFMT training programs and timing. One RCT involving 107 patients found that PFMT led to a significantly reduced likelihood of experiencing UI at 1, 3, 6, and 12 months compared to no treatment, with progressively increasing continence rates in the treatment group [34]. However, there was a differential dropout bias, as the control group had a dropout rate of 24.5% compared to no dropouts in the treatment group, which was not adequately addressed. Overall, a Cochrane review and meta-analysis from 2015 could not conclude any long-term benefit of PFMT based on the current available literature [35]. Interestingly, only four centers in our study offered PFMT preoperatively. An RCT by Centermero et al. involving 118 patients found that preoperative PFMT improved early continence and quality of life compared to postoperative PFMT alone [12]. However, this finding could not be replicated in an RCT by Gerearts et al., which involved 180 patients [13]. In their study, initiating PFMT preoperatively made no difference compared to postoperative PFMT only. A systematic review and meta-analysis by Chang et al. concluded, based on available literature, that there is an improvement in early continence with preoperative PFMT [36]. Some centers in our study offered PFMT only if patients had UI problems, although the specific criteria for deeming patients to have UI problems were not described. It is unlikely that PFMT would be beneficial to patients who still experience UI after one year, as concluded in a quasi-randomized controlled trial [33]. One center in our study included medical intervention with duloxetine. However, the use of duloxetine in continence rehabilitation is not recommended by international guidelines, as it has shown a positive effect on early continence but not on long-term continence, and discontinuation due to adverse effects is common [37, 38].

The main strength of our study is the relatively high response rate compared to previous reports. However, our response rate of 64.3% does pose a risk of non-response bias in our results, potentially limiting the accuracy and generalizability of the findings. The most likely effect of this would be an overestimation of the thoroughness of follow-up and rehabilitation as centers offering these could be most likely to take the time to respond to a survey. Due to the nature of the survey, our open-ended questions led to differences between centers in the level of detail provided in the description of rehabilitation protocols. In this regard, we enquired about institutional practices on both functional evaluations and rehabilitation and our survey was not designed to capture variations in the practices of individual healthcare personal within each institution. Additionally, the study aimed to investigate the practices in the Nordic countries, which means that the findings cannot be generalized beyond this specific area. To the authors’ knowledge, the management of cancer Nordic patients were not changed due to the COVID-19 pandemic, however, due to the timing of the study it cannot be completely excluded that the reported management reflected adjusted strategies in some centers.

In conclusion, significant variations exist in the clinical practices of centers in the Nordic countries regarding the evaluation of sexual and urinary function in relation to RP and the implementation of rehabilitation protocols. There is a clear need for standardized evaluation protocols, including guidelines on the specific PROMs to be used, the timing of evaluations, and the duration of assessment periods. Similarly, there seem to be a need for an increased focus on sexual rehabilitation with utilization of options other than a simple PDE5I. These efforts will contribute to improved consistency and quality in the management of sexual and urinary function after RP.

### Supplementary information

Questionnaire

## Data Availability

The dataset generated during the current study are available from the corresponding author on reasonable request.
