# Supplementary material for: Management of functional outcomes after radical prostatectomy in the Nordic countries: A survey of uro-oncological centers
Source: Int J Impot Res. 2023 Oct 10;36(5):486–92. doi: 10.1038/s41443-023-00772-8 (PMC11251983; doi:10.1038/s41443-023-00772-8)
Supplement: Supplementary file 1 — Questionnaire [file 41443_2023_772_MOESM1_ESM.docx]

**Post-prostatectomy rehabilitation: a systematic evaluation of clinical practice in the Nordic countries.**

Questionnaire

How many radical prostatectomies are performed at your center each year? _________________________

How is the distribution between surgical approaches?

Open prostatectomies _______%

Robot assisted prostatectomies _______%

Conventional laparoscopic prostatectomies _______%

Do you evaluate the patients’ urinary function before surgery? Yes / no

How is this done (see questions below)?

Objectively? Yes / no

If “yes”, please explain ___________________________________________________________________________________________

___________________________________________________________________________________________

Validated questionnaire(s)? Yes / no

If “yes”, please explain ___________________________________________________________________________________________

___________________________________________________________________________________________

Non-validated questionnaire(s)? Yes / no

If “yes”, please explain ___________________________________________________________________________________________

___________________________________________________________________________________________

Subjective assessment? Yes / no

If “yes”, please explain – who performs the assessment? ___________________________________________________________________________________________

___________________________________________________________________________________________

Other methods? ___________________________________________________________________________________________

___________________________________________________________________________________________

Do you evaluate the patients’ sexual function before surgery? Yes / no

How is this done (see questions below)?

Objectively? Yes / no

If “yes”, please explain ___________________________________________________________________________________________

___________________________________________________________________________________________

Validated questionnaire(s)? Yes / no

If “yes”, please explain ___________________________________________________________________________________________

___________________________________________________________________________________________

Non-validated questionnaire(s)? Yes / no

If “yes”, please explain ___________________________________________________________________________________________

___________________________________________________________________________________________

Subjective assessment? Yes / no

If “yes”, please explain – who performs the assessment? ___________________________________________________________________________________________

___________________________________________________________________________________________

Other methods? ___________________________________________________________________________________________

___________________________________________________________________________________________

Do you evaluate the patients’ urinary function after surgery? Yes / no

When is the evaluation performed (eg. 3, 6 and/or 12 months after surgery)?

How is the evaluation done (see questions below)?

Objectively? Yes / no

If “yes”, please explain ___________________________________________________________________________________________

___________________________________________________________________________________________

Validated questionnaire(s)? Yes / no

If “yes”, please explain ___________________________________________________________________________________________

___________________________________________________________________________________________

Non-validated questionnaire(s)? Yes / no

If “yes”, please explain ___________________________________________________________________________________________

___________________________________________________________________________________________

Subjective assessment? Yes / no

If “yes”, please explain – who performs the assessment? ___________________________________________________________________________________________

___________________________________________________________________________________________

Other methods? ___________________________________________________________________________________________

___________________________________________________________________________________________

Do you evaluate the patients’ sexual function after surgery? Yes / no

When is the evaluation performed (eg. 3, 6 and/or 12 months after surgery)?

How is the evaluation done (see questions below)?

Objectively? Yes / no

If “yes”, please explain ___________________________________________________________________________________________

___________________________________________________________________________________________

Validated questionnaire(s)? Yes / no

If “yes”, please explain ___________________________________________________________________________________________

___________________________________________________________________________________________

Non-validated questionnaire(s)? Yes / no

If “yes”, please explain ___________________________________________________________________________________________

___________________________________________________________________________________________

Subjective assessment? Yes / no

If “yes”, please explain – who performs the assessment? ___________________________________________________________________________________________

___________________________________________________________________________________________

Other methods? ___________________________________________________________________________________________

___________________________________________________________________________________________

Are there published functionel results from your center? Yes / no

Do you have a rehabilitation program for continence? Yes / no

If ”yes” please describe:

___________________________________________________________________________________________

___________________________________________________________________________________________

Do you have a rehabilitation program for sexual function? Yes / no

If ”yes” please describe:

___________________________________________________________________________________________

___________________________________________________________________________________________

Who is offered the continence rehabilitation?

All patients ___

Only those who receive nerve sparing surgery ___

Other restrictions? Yes / no

If ”yes” please describe:

___________________________________________________________________________________________

___________________________________________________________________________________________

Who is offered the sexual rehabilitation?

All patients ___

Only those who receive nerve sparing surgery ___

Other restrictions? Yes / no

If ”yes” please describe:

___________________________________________________________________________________________

___________________________________________________________________________________________

Is it your impression that continence rehabilitation improves the long term continence of patients at your center? Yes / No

Is it your impression that sexual rehabilitation improves the long term continence of patients at your center? Yes / No
